# The Cardiologist Driving Synthetic AI: The TIMA Method for Clinically Supervised Synthetic Data Generation

**DOI:** 10.3390/jcm15041351

**Published:** 2026-02-09

**Authors:** Gianmarco Parise, Roberto Ceravolo, Fabiana Lucà, Michele Massimo Gulizia, Cecilia Tetta, Orlando Parise, Federico Nardi, Massimo Grimaldi, Sandro Gelsomino

**Affiliations:** 1 CARIM, School for Cardiovascular Diseases, Maastricht University, 6211 LK Maastricht, The Netherlands; 2 Department of Cardiology, Lamezia Terme Hospital, 88046 Lamezia Terme, Italy; 3 Epidemiology Area, Italian Association of Hospital Cardiologists (ANMCO), 50121 Firenze, Italy; 4 Department of Cardiology, Garibaldi-Nesima Hospital Catania, 95122 Catania, Italy; 5 Independent Researcher, 40137 Bologna, Italy; 6 Independent Researcher, 87100 Cosenza, Italy; 7 Department of Cardiology, S. Spirito Hospital, 15033 Casale Monferrato, Italy; 8 Department of Cardiology, Acqua Viva Delle Fonti Hospital, 70021 Bari, Italy; 9 Cardiovascular Research Institute Maastricht (CARIM), Maastricht University, 6211 LK Maastricht, The Netherlands

**Keywords:** synthetic artificial intelligence, cardiology, clinical validation, TIMA, predictive models

## Abstract

**Background/Objectives:** Synthetic artificial intelligence (AI) is increasingly used in cardiovascular medicine to generate realistic clinical data from limited samples while preserving patient privacy. Despite its promise, concerns remain regarding the clinical reliability of synthetic datasets, which hampers their integration into routine practice. This article introduces the TIMA method (Team-Implementation Multidisciplinary Approach), designed to involve clinicians directly in every phase of synthetic data development. The objective of this work is to describe the TIMA framework and to illustrate how structured clinician–data scientist collaboration can enhance the clinical robustness and plausibility of synthetic AI outputs. **Methods:** The TIMA approach structures the synthetic data generation workflow around continuous interaction between clinicians and data scientists. Cardiologists define clinical constraints, verify inter-variable relationships, and assess the coherence and plausibility of generated records. The framework is illustrated through multiple cardiology use cases, including atrial fibrillation risk prediction and surgical mortality estimation in infective endocarditis, to demonstrate its adaptability across different clinical contexts. Each phase includes iterative validation steps aimed at ensuring alignment with established clinical knowledge rather than reporting quantitative performance outcomes. **Results:** Application of the TIMA framework supported the development of synthetic datasets that adhered more closely to clinical logic and domain-specific constraints. Clinician–data scientist collaboration enabled early detection of implausible variable interactions, improved interpretability of synthetic data patterns, and enhanced internal consistency across different cardiology-oriented scenarios. **Conclusions:** TIMA represents a scalable and replicable methodological model for integrating synthetic AI into cardiology by embedding clinical expertise throughout the data generation process. Its structured, multidisciplinary workflow supports the production of synthetic data that is not only statistically coherent but also clinically meaningful, thereby strengthening trust and reliability in AI-assisted cardiovascular research.

## 1. Introduction

Artificial intelligence (AI) is rapidly transforming modern medicine, enhancing the decision-making, predictive, and diagnostic capabilities of clinical systems. Among its many branches, synthetic AI, defined as the generation of realistic clinical data using generative models such as Generative Adversarial Networks (GANs), represents one of the most promising approaches to overcome the limitations of real-world clinical datasets, including data scarcity, heterogeneity, ethical constraints, and privacy concerns [1].

Cardiology, with its inherently data-driven nature and continuous production of structured data, signals, images, and time series, is particularly well suited to the adoption of synthetic AI. Nevertheless, its clinical use remains limited, largely due to persistent concerns regarding the reliability and trustworthiness of synthetic data [2]. This lack of trust is not merely theoretical: AI models are often developed within predominantly technical environments, with limited clinical involvement, resulting in outputs that—despite technical sophistication—may be poorly aligned with real-world clinical reasoning and practice [3].

Recent literature consistently indicates that the perceived reliability of synthetic data strongly depends on the systematic integration of clinicians throughout the generative process [4]. In the absence of active clinical supervision, generative models may produce incoherent, implausible, or weakly representative patient profiles, thereby limiting their practical utility and clinical acceptance. A recent review highlights that clinician trust in synthetic AI requires transparent, verifiable, and clinically supervised development frameworks [5]. Recent comprehensive reviews and expert analyses published in 2024 and 2025 further reinforce these concerns. In particular, recent surveys of synthetic medical data generation and evaluation report that current assessment practices often emphasize fidelity/utility/privacy and highlight missing or insufficient clinical validation, together with a lack of standardized, clinically relevant evaluation benchmarks [6,7,8].

Importantly, recent position papers explicitly identify the lack of structured and continuous clinical supervision as a major barrier to the real-world adoption of synthetic data for clinical AI [9]. This limitation has become even more relevant with the emergence of large language models for synthetic clinical data generation, which may amplify the risk of producing clinically plausible yet misleading patient profiles in the absence of expert validation [9].

Within this context, the TIMA method (Team-Implementation Multidisciplinary Approach) has been developed as an innovative strategy that places cardiologists at the core of the synthetic AI lifecycle, from the definition of clinically grounded rules to the final validation of generated data. The aim of this article is to describe the TIMA framework, illustrate its practical application, and discuss its implications for contemporary cardiology. For synthetic data to become a reliable and widely adoptable tool in clinical practice, they must fulfill requirements that go well beyond statistical quality alone. Semantic coherence, adherence to clinical guidelines, internal clinical logic, traceability of rules, and independent validation are only some of the elements considered indispensable [6,9]. However, a recent systematic review has shown that most generative models applied in medicine overlook these aspects and focus almost exclusively on computational performance.

This situation is further complicated by the lack of shared standards on what constitutes “good” synthetic data in healthcare. In the absence of clinically meaningful metrics, each study evaluates results with different criteria, making it difficult to compare approaches and to identify best practices. A recent scoping review of reviews has highlighted this fragmentation, calling for the urgent definition of guidelines and uniform criteria for the evaluation of synthetic datasets, especially in high-impact fields such as cardiology [7].

The aim of this article is to introduce and formalize a clinically governed methodological framework for synthetic data generation in cardiology, while empirical performance evaluation and dataset-specific analyses are intentionally left to future studies.

## 2. Materials and Methods: The TIMA Framework

The TIMA method (Team-Implementation Multidisciplinary Approach), first introduced by Gelsomino and Parise [4], represents an innovative clinical–technical model in which the cardiologist has a central and continuous role in the development of synthetic AI-based data. Unlike traditional paradigms, TIMA requires the active participation of clinicians throughout the entire process, from the definition of clinical rules to the final validation of coherence and plausibility of the generated data. All figures are based on synthetic datasets generated from real-world clinical data and are intended to illustrate the methodological principles of the TIMA framework rather than to report quantitative experimental results.

**Figure 1:**
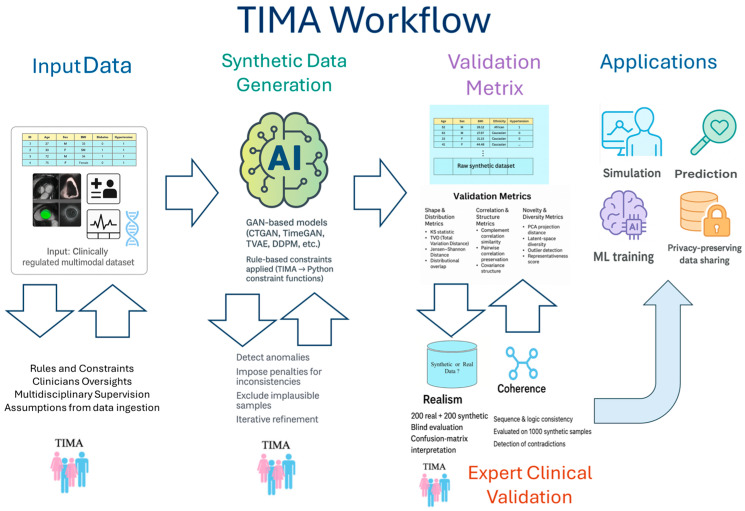
Operational structure of TIMA, showing multidisciplinary definition of clinical constraints, a continuous clinician–developer feedback loop, and phased validation of synthetic data for coherence, usability, and clinical plausibility.

To visually clarify this framework, Figure 1 illustrates the operational structure of TIMA. The process begins with the definition of clinical constraints by a multidisciplinary team, followed by a continuous feedback loop between clinicians and developers. Subsequent phases involve the validation of generated data, with particular attention to internal coherence, usability, and clinical adherence. This model allows medical reasoning to be embedded directly into generative algorithms, ensuring that synthetic data are not only statistically valid but also clinically plausible.

### 2.1. Composition of the Clinical Team: A Pathology-Adaptable Model

In the original work, the TIMA committee consisted of a cardiologist, a cardiac surgeon, an anesthesiologist, and an epidemiologist, reflecting the infectious and in-hospital context of the COVID-19 pandemic [4]. However, the composition of the TIMA team is not fixed; rather, it adapts to the specific disease or clinical area under study. In cardiology, the structure of the group can be modulated according to the objective of the generative model. Examples include:

- **Heart failure.** The multidisciplinary team should include a cardiologist specialized in heart failure, a specialized heart failure nurse, and a clinical pharmacologist. This composition is recommended to ensure integrated and specialized patient management [10].
- **Atrial fibrillation.** For the management of this complex arrhythmia, the team should involve an electrophysiologist, a cardiac surgeon, a clinical cardiologist, and imaging specialists. Such a group can provide a structured diagnostic and therapeutic pathway [11].
- **Cardiovascular prevention and risk stratification.** In this setting, the ideal team includes a cardiologist experienced in prevention, an endocrinologist, and an internist specialized in metabolic syndrome. The multidisciplinary approach improves risk assessment and treatment adherence [12].
- **Transcatheter aortic valve implantation (TAVI).** For TAVI procedures, the so-called “Heart Team” includes an interventional cardiologist, a cardiac surgeon, an anesthesiologist, an echocardiographer, and, in more complex cases, a geriatrician. The presence of complementary professionals enables comprehensive assessment of eligibility and procedural risk [13].

These examples show how the flexibility of team composition represents one of the main strengths of the TIMA method, allowing clinically relevant supervision tailored to the specific domain of application. The guiding principle remains unchanged: each team member operates independently of the technical development, maintaining a purely clinical perspective that is not influenced by modeling or engineering goals.

In several real-world clinical settings, limitations related to data availability, heterogeneity, and governance still represent a significant barrier to the development of clinically reliable AI models. The integration of TIMA into these settings can therefore leverage existing expertise and collaborative dynamics, facilitating the adoption of innovative synthetic data generation and validation models [14].

### 2.2. First Clinical Evaluation Phase: Testing Model Learning

This phase represents an internal methodological step within the TIMA framework and does not aim to report experimental results, but rather to assess the alignment between model learning behavior and clinically expected patterns. Before definitive data generation, the TIMA committee receives an initial synthetic dataset to evaluate whether the model has correctly learned essential clinical constraints. At this stage, the cardiologist reviews generated clinical variables and flags incoherences, missing dependencies, or contradictory clinical relationships. For example, the clinician may detect implausible drug combinations or inconsistent clinical trajectories. These observations guide the implementation of binding rules to correct identified anomalies.

This initial step is crucial to prevent the automatic generation of clinically implausible patient profiles. Recent work shows that **human expert oversight and iterative validation**—including early-stage checks for requirement satisfaction and clinical accuracy, improves the reliability of generative outputs [15,16].

Moreover, clinical supervision can help incorporate complex semantic relationships that purely statistical checks may miss, highlighting the need for clinician-grounded evaluation pipelines [9,16].

Early identification of “unlearned” variables can also support the design of customized constraint functions that improve coherence across dataset components. Systematic evaluation frameworks and the lack of consensus on best-practice metrics are repeatedly identified as central issues for trust and adoption in medical synthetic data [17].

### 2.3. Integration of Clinical Rules Into Generative Models

Once the TIMA committee has identified inconsistencies in the initial synthetic dataset, clinical observations are translated into binding rules that guide the behavior of the generator during subsequent training cycles. The cardiologist, in collaboration with the technical team, contributes to the formulation of these rules by translating medical logic into structured constraints that can be implemented in software.

Within the TIMA framework, clinical rules can be operationalized, for example, through Python (Python v3.11, preferred for full package compatibility)-based implementations within frameworks such as the **Synthetic Data Vault (SDV)**, as an illustrative technical realization of the proposed constraint-based workflow.

**Figure 2:**
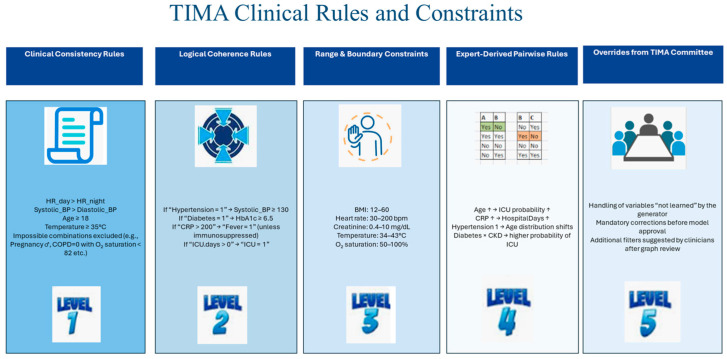
Categories of clinical rules in TIMA, including clinical consistency, logical coherence, range/boundary constraints, expert-derived pairwise rules, and committee overrides to handle edge cases, illustrated with representative examples.

Figure 2 summarizes the main categories of clinical rules adopted within the TIMA framework. Clinical consistency rules enforce basic physiological and clinical plausibility constraints, logical coherence rules encode conditional dependencies among variables, range and boundary constraints define admissible value intervals, and expert-derived pairwise rules capture clinically meaningful associations between variables. The final level represents expert overrides applied by the TIMA committee to address edge cases or generator blind spots. The examples reported in the figure are representative and intended to illustrate how each category of rules can be operationalized in practice. Typical examples include:

1. **Procedural constraints**, such as preventing the generation of intubated patients who are not admitted to an intensive care unit.
2. **Therapy–context incompatibilities**, for instance, the clinically discouraged combination of chronic obstructive pulmonary disease (COPD) with non-selective beta-blockers, given their potential bronchoconstrictive effects.
3. **Exclusion of conflicting therapeutic combinations**, where two mutually exclusive drugs cannot be assigned to the same patient.

Although Figure 2 provides a conceptual overview, each rule category is illustrated by concrete, clinically motivated examples that can be translated into formal constraints (e.g., logical conditions, inequalities, or pairwise dependencies) depending on the target implementation.

The application of clinical rules improves not only the internal coherence of the data but also their acceptability among the medical community. Recent experiences in oncology and pharmacology show that early integration of clinical knowledge improves realism metrics and the predictive performance of downstream models [18,19]. In cardiovascular medicine, the incorporation of pathophysiological and therapeutic constraints has been particularly useful for maintaining a balance between statistical coherence and clinical plausibility, especially in highly complex decision-making contexts such as advanced heart failure and thromboembolic risk management [20]. The literature confirms that the clinical usefulness of synthetic data strongly depends on the model’s ability to respect medical causal rules that are not automatically learned by algorithms but must be formalized through expert input [21].

### 2.4. Structural Assessment: Analysis of Statistical–Clinical Congruence

After controlled generation, the TIMA team proceeds to a structural assessment of the synthetic dataset, comparing relationships between variables with those observed in the original data. This phase is crucial to ensure that clinical constraints are respected not only at the individual level but also at the global level, preserving realistic statistical structures and clinically meaningful patterns.

In particular, the committee analyzes:

(a) **Univariate distributions**, that is, the frequency of each clinical variable (e.g., age, blood pressure, creatinine) in the synthetic dataset compared with the real dataset;
(b) **Bivariate distributions**, that is, the joint behavior of pairs of associated clinical variables (e.g., age and prescription of anticoagulants), to verify that expected clinical associations are preserved in the synthetic data;
(c) **Coherence of correlations between pairs of numerical variables** (e.g., renal function and use of nephrotoxic drugs);
(d) **Frequency of clinical combinations** (e.g., atrial fibrillation, advanced age, and anticoagulant use occurring together);
(e) **Alignment with patterns observed in the original dataset**.

This assessment uses automated plots, contingency tables, heatmaps, and synthetic indicators such as contingency scores and correlation similarity scores, with predefined thresholds of acceptability (e.g., median ≥ 0.9) before proceeding to the next phase. Figure 2 provides exemplary comparisons between real and synthetic data used to evaluate internal coherence, clinical plausibility, and adherence to known pathophysiological relationships.

The TIMA approach differs from purely numerical validation methods because it combines quantitative measures with expert clinical judgment to determine whether observed patterns are not only statistically sound but also clinically credible [22]. Recent experiences in healthcare have demonstrated that integrated clinical–statistical validation significantly enhances the robustness of synthetic datasets and reduces the risk of artificial plausibility, that is, the generation of patterns that are statistically correct but clinically misleading [8]. Structural validation is therefore a key guarantee of clinical reliability and a prerequisite for the use of synthetic datasets in research, simulation, or algorithm training.

### 2.5. Final Validation: Clinical Realism and Individual Coherence

The final phase of the TIMA method consists of an independent clinical validation of the synthetic dataset to test its realistic adherence to medical reasoning and the internal coherence of individual cases. In practice, **within illustrative applications of the TIMA framework**, the TIMA committee may receive a random mixed sample on the order of one to a few hundred real and synthetic cases (e.g., approximately 100–200 per group) presented without labels. Team members, including the cardiologist, evaluate the clinical plausibility of each case, providing a binary judgment (plausible/non-plausible) or a rating on a scale. The objective is to assess whether synthetic data are indistinguishable from real data in the eyes of clinical experts.

In parallel, a larger subset of the synthetic dataset (for example, 1000 cases) undergoes systematic internal coherence checks, ensuring that each record maintains consistent clinical logic across all variables. For instance, patients treated with anticoagulants must present at least one justifying clinical condition (e.g., atrial fibrillation, venous thromboembolism, or prosthetic valves).

In a separate illustrative application of the TIMA framework, conducted outside the scope of the present manuscript, a blinded clinical validation procedure involving on the order of one to a few hundred real and synthetic cases has been performed. Notably, Parise et al. reported a blinded expert assessment in which synthetic cases were recognised as authentic by nearly 100%, supporting the feasibility of this type of expert-driven validation within TIMA-like workflows [4].

This validation approach, grounded in expert judgment, is inspired by methodologies used to assess diagnostic interfaces and decision aids in real clinical workflows, such as the DDX-BRO cluster-randomized trial evaluating a computerized diagnostic decision support system in emergency patients [23]. Multiple studies confirm that clinical realism is essential for the adoption of synthetic data in practice and can be more influential than statistical metrics alone [2,5].

Without this qualitative verification, synthetic datasets risk remaining confined to experimental settings, unable to gain the trust of clinicians and healthcare decision-makers.

### 2.6. Data Synthesis Process Within the TIMA Framework

Within the TIMA framework, data synthesis is conceived as a structured, multi-step process rather than as a single model-driven operation. First, real-world clinical data undergo standard preprocessing steps, including variable selection, harmonization, and basic quality checks, to ensure internal consistency and clinical interpretability. Second, a generative model appropriate to the data modality is employed to produce an initial synthetic dataset. At this stage, the focus is on capturing global statistical patterns rather than enforcing strict clinical constraints.

Subsequently, clinician-guided rules and constraints are integrated into the synthesis pipeline. These rules encode clinical knowledge in the form of plausibility conditions, logical dependencies, range limitations, and expert-defined associations among variables. The resulting synthetic data are then subjected to iterative validation, during which clinical experts review representative samples to assess plausibility and coherence. Feedback from this validation step is used to refine rules and constraints, yielding progressively improved synthetic datasets. This process-oriented description emphasizes the logic and governance of data synthesis within TIMA, independent of specific algorithms or software implementations.

## 3. Use Cases

The following clinical applications represent the practical outcomes of the TIMA framework, illustrating its use in real-world cardiology scenarios rather than reporting conventional experimental results.

### 3.1. Transition to Illustrative Cardiovascular Use Cases

To clarify how TIMA can be applied beyond the methodological framework described above, we present two cardiovascular use cases. These examples are not intended as clinical studies or outcome analyses, but as conceptual demonstrations of how TIMA-validated synthetic datasets can support different modelling tasks in cardiology. Their purpose is to illustrate the practical applicability, flexibility, and clinical reasoning embedded in TIMA, rather than to provide clinical evidence.

#### 3.1.1. Illustrative Use Case 1—Atrial Fibrillation Risk Modeling Using TIMA-Validated Synthetic Data

A first illustrative example shows how the TIMA framework can be applied to generate clinically coherent cardiac time-series for atrial fibrillation (AF). In this context, TIMA ensures that the generative model respects physiological constraints—such as circadian variation in heart rate, realistic heart-rate variability dynamics, or plausible transitions toward arrhythmic patterns—before any synthetic sequence is accepted as usable. The resulting dataset is not intended to reproduce a specific clinical study, but to demonstrate how the mechanisms described in Section 2 and Section 3 translate into real operational decisions: definition of medical rules, supervised refinement of the generator, and structured validation combining statistical metrics with expert judgment.

**Figure 3:**
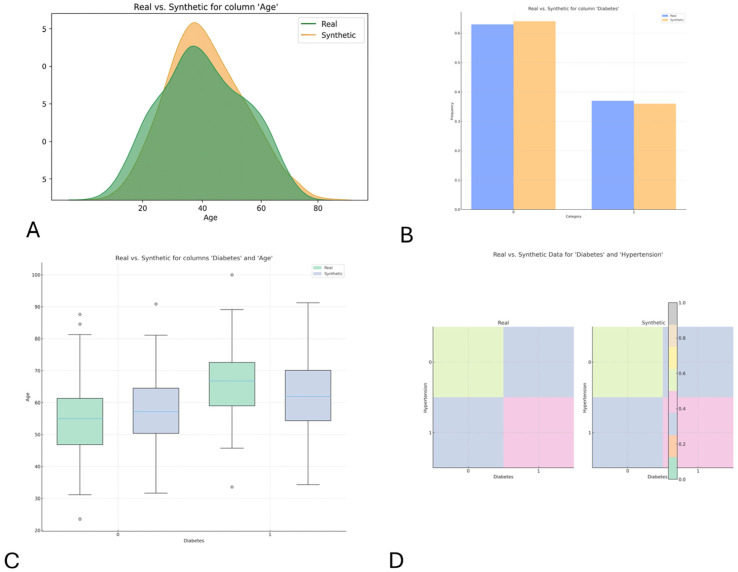
Realism between real and synthetic data. (**A**) Continuous Variable. (**B**) Discrete Variable. (**C**) Discrete vs. Continuous Variable. (**D**) Heat map of the comparison between discrete Variables.

The distributional comparisons presented in Figure 3A–D illustrate how TIMA may potentially verify that synthetic sequences reproduce the essential characteristics of the source data without collapsing into unrealistic patterns. This example shows why TIMA is particularly valuable when modeling physiological trajectories, which are highly sensitive to artifacts and require strong clinical oversight to remain interpretable. The AF case therefore serves as a conceptual demonstration of how TIMA can prepare synthetic time-series suitable for exploratory simulation, prototype risk-modeling or early feasibility testing.

#### 3.1.2. Illustrative Use Case 2—Modeling Surgical Risk in Infective Endocarditis Using TIMA

In the second illustrative example, TIMA is applied to the generation of synthetic clinical scenarios relevant to embolic risk modeling in infective endocarditis. Rather than functioning as a retrospective filter, TIMA operates throughout the generative process, progressively steering the model toward patterns that reflect real clinical behavior. This is particularly important in endocarditis, where crucial high-risk phenotypes—such as prosthetic valve infection, septic emboli, or hemodynamic instability—are frequently underrepresented in real-world registries.

**Figure 4:**
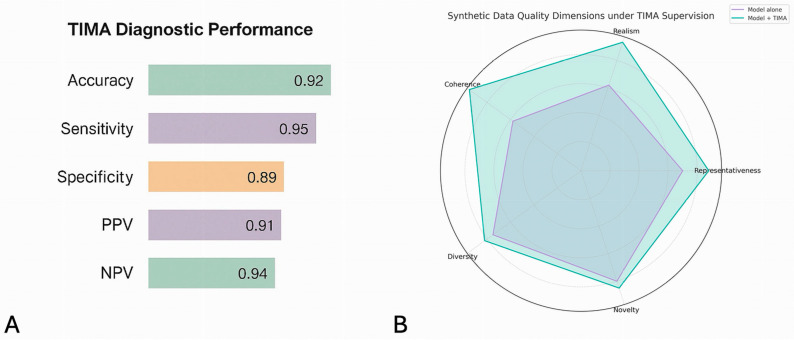
As shown for Diagnostic performance (**A**) and Data quality (**B**).

Figure 4 shows how TIMA’s realism and coherence may potentially enable synthetic scenarios to converge toward clinically recognizable trajectories. Relationships between pathogen type, valve involvement, inflammatory markers, and embolic vulnerability are preserved, while still allowing controlled variability across patient profiles. This gradual alignment is essential in a condition as complex as endocarditis, where pathophysiological interactions are non-linear and strongly dependent on the clinical context. Beyond expert perception, TIMA also produces measurable improvements in structural quality.

**Figure 5:**
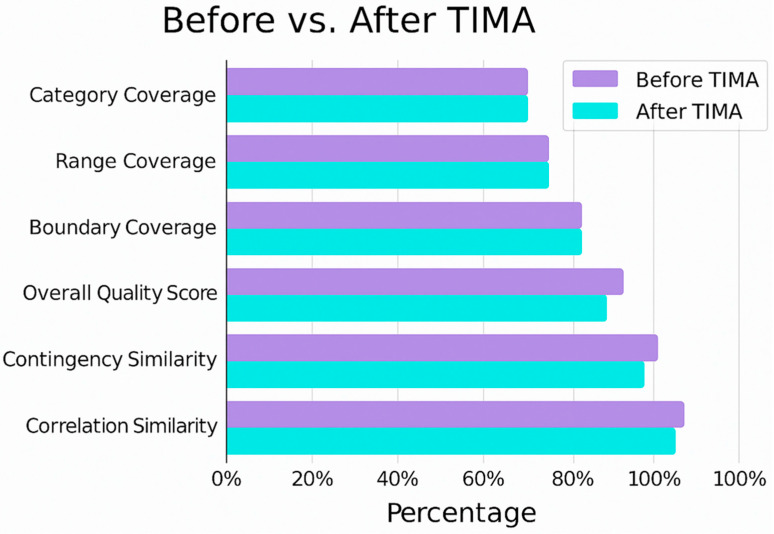
Graph of synthetic data overall performance.

As illustrated in Figure 5, key quantitative indicators, such as distributional fidelity, correlation preservation, and representativeness, consistently improve after TIMA-guided refinement. These gains demonstrate that clinically grounded rules do not merely “clean” the synthetic dataset but actively enhance its capacity to support modeling tasks. Taken together, this example shows how TIMA transforms synthetic scenario generation into a medically guided process, yielding datasets sufficiently realistic and structurally coherent to support embolic-risk analysis in infective endocarditis.

The number of clinical experts involved in the TIMA validation process is not fixed and can be adapted to the clinical context. In illustrative applications, validation may be performed by a small panel of domain experts (e.g., 2–5 clinicians). Agreement among reviewers can be assessed using simple consensus rules or standard inter-rater agreement measures (e.g., percentage agreement or kappa statistics), depending on the objectives of the validation. Consideration of inter-rater variability is an integral part of the TIMA framework design, although formal agreement analysis is not reported in the present methodological study.

## 4. Discussion

The use of synthetic AI is rapidly evolving within hospital cardiology. Initially conceived as a response to regulatory constraints related to privacy and data scarcity, synthetic AI is increasingly emerging as a tool for active knowledge generation, able to simulate complex clinical scenarios and support predictive models even in highly variable contexts. In cardiovascular medicine, applications range from digital twins in heart failure and electrophysiology to the simulation of procedural decisions in transcatheter coronary and structural interventions, including transcatheter aortic valve implantation (TAVI/TAVR) [24].

One of the main obstacles to the widespread adoption of synthetic AI in medicine, however, remains its perceived reliability, that is, the degree of trust clinicians place in generated data. Many professionals are reluctant to rely on datasets that do not derive from direct clinical observation, fearing that these data may be incoherent, artificial, or disconnected from real-world practice [2]. This cultural barrier is reinforced by the fact that many synthetic models are developed in purely computational environments, without meaningful involvement of healthcare professionals in their design and validation.

### 4.1. Overcoming the Trust Barrier: The Role of Clinical Participation

To address this structural limitation, we introduced the TIMA method, which proposes a radical change in perspective: within the TIMA framework, clinicians act as active contributors to the supervision, validation, and iterative refinement of synthetic data, rather than passive end-users of automatically generated outputs. By involving clinicians at each step, TIMA transforms synthetic data into a shared clinical–technical product, whose logic and provenance are transparent to the end users.

This shift directly targets the trust barrier. Data that have passed the scrutiny of a TIMA committee are perceived as clinically valid and usable because they have been generated according to logics that clinicians understand and endorse. As shown in other clinical domains, clinician engagement in co-design and governance increases acceptance and adoption of digital tools in routine practice [25,26,27].

### 4.2. A New Form of Clinical–Technical Interaction

TIMA promotes a horizontal collaboration between clinicians and developers, in which both groups share responsibility for data creation. This approach breaks down disciplinary silos and opens the way to a new role for physicians: not only users of models but also designers of clinical data, responsible for their plausibility and usability.

This concept, which can be described as clinical data stewardship, parallels recent approaches in computational medicine, where direct clinician involvement in the data lifecycle is considered essential for ensuring output quality and safety [4]. In this sense, TIMA provides a concrete operationalization of data stewardship, aligning model design with clinical reasoning from the outset.

### 4.3. A Concrete Educational Opportunity

The TIMA method also creates a structured environment for bidirectional learning. On the one hand, clinicians acquire foundational knowledge about synthetic data generation and validation. On the other hand, engineers and data scientists gain a deeper understanding of clinical logic, pathophysiological constraints, and the practical implications of health variables.

This cross-training is far from marginal. In this era, in which AI models increasingly enter daily clinical practice, hospital physicians require advanced digital literacy, and data scientists need a solid understanding of clinical concepts [28]. The TIMA experience demonstrates that this exchange is not only feasible but highly productive, resulting in models that are both technically robust and clinically meaningful.

### 4.4. A Replicable Path to Standardization

The value of the TIMA method goes beyond the individual projects in which it has been implemented. Its modular and repeatable structure makes it a potentially standardizable model for any clinical specialty aiming to integrate expert supervision into AI workflows.

In our group, TIMA is being applied in very different contexts, from atrial fibrillation prediction to estimation of surgical mortality in infective endocarditis, demonstrating adaptability, rigor, and reproducibility. International studies increasingly emphasize the need for “expert clinical validation frameworks” as a prerequisite for deploying AI in healthcare. TIMA fits precisely within this emerging requirement.

The method can be readily replicated in other areas such as oncology, nephrology, and emergency medicine, where synthetic data are promising but still hindered by limited methodological transparency. In these fields, the introduction of an independent clinical committee with structural decision-making power offers an effective strategy to balance innovation with clinical responsibility.

### 4.5. Ethical and Legal Considerations

The use of synthetic data in clinical contexts raises important ethical and legal considerations, particularly when such data are intended to support clinical decision-making or downstream machine learning models. While clinician involvement in the generation and validation process improves clinical fidelity and plausibility, it may also influence how synthetic “patients” are represented. Without appropriate governance, there is a risk that expert supervision could inadvertently introduce normative or idealized representations of patients rather than faithfully reflecting real-world clinical heterogeneity.

Within the TIMA framework, clinician involvement is explicitly constrained by clinical guidelines, predefined rules, and transparent validation procedures, with the goal of reducing subjectivity and ensuring accountability. Expert input is used to enforce plausibility and consistency, not to optimize outcomes or impose preferred clinical profiles. In this sense, TIMA is designed to enhance ethical robustness by making human oversight explicit, auditable, and rule-based, rather than implicit or opaque. Nevertheless, we acknowledge that the ethical and legal implications of synthetic data use require continuous scrutiny, particularly with respect to data provenance, representativeness, and responsibility for downstream use. For this reason, TIMA emphasizes governance structures as a core component of clinically responsible synthetic data generation.

### 4.6. Bias, Rare Diseases Bias, Rare Diseases, and Limitations

A key limitation of synthetic data generation, particularly relevant for rare or low-prevalence diseases, is the potential amplification of existing biases present in the source data. When real-world datasets contain limited or unbalanced representations of specific clinical subgroups, generative models may reproduce or even reinforce these distortions, leading to synthetic cohorts that inadequately reflect rare conditions or atypical patient profiles. While synthetic data can partially mitigate data scarcity, they do not inherently resolve issues related to underrepresentation.

Within the TIMA framework, this risk is addressed through continuous clinical supervision, expert-defined rules, and iterative validation procedures aimed at preserving clinical plausibility and guideline consistency. In particular, clinician involvement allows the identification of implausible patterns, unrealistic distributions, or systematic omissions affecting rare disease phenotypes. Nevertheless, we acknowledge that no synthetic data generation approach can fully eliminate bias, especially in settings characterized by extreme data sparsity. For this reason, the use of synthetic data in rare-disease contexts should be accompanied by careful monitoring, transparency regarding data provenance, and cautious interpretation of downstream model performance.

This work is intentionally positioned as a methodological and conceptual validation of the TIMA framework rather than as a fully specified software or benchmarking study. While the framework has already been applied to concrete clinical modeling tasks—including time-series generation, tabular risk modeling, and outcome prediction—those implementations are beyond the scope of the present manuscript. Here, the objective is to formalize and validate the conceptual logic, governance structure, and clinical integration principles underlying TIMA. Model-specific choices, hyperparameter configurations, and mathematical formulations are therefore deliberately abstracted to preserve generality and to avoid overfitting the framework to a single data modality or clinical task. Dedicated implementation-focused studies are currently ongoing and will report detailed architectures, optimization strategies, and empirical benchmarks.

### 4.7. Clinical Utility

From a clinical perspective, the TIMA framework addresses the critical “trust gap” that has so far limited the adoption of synthetic AI in cardiovascular medicine. By embedding clinician-defined constraints directly into the generative process, TIMA aims to ensure that the resulting data are not only statistically plausible, but also pathophysiological coherent and clinically interpretable. This has clear relevance for privacy-preserving clinical simulation, for the development and stress-testing of risk-prediction models (e.g., in atrial fibrillation or infective endocarditis), and for digital-twin-oriented research workflows, while maintaining strict safeguards for patient privacy. Ultimately, the clinical value of TIMA lies in enabling high-quality, expert-vetted synthetic datasets that can support the safe development, validation, and dissemination of AI-driven diagnostic and therapeutic tools in cardiology.

## 5. Conclusions

Synthetic AI is an emerging resource for modern cardiology, with the potential to improve clinical prediction, optimize resource allocation, and support personalized care. However, full exploitation of this potential requires models that are not only statistically accurate but also reliable and acceptable from a clinical standpoint.

The TIMA method, described here and applied in real-world contexts, offers a structured solution to this need. By actively involving clinicians throughout the entire lifecycle of synthetic data—from rule definition to final validation—TIMA turns data generation into a shared, verifiable, and clinically anchored process.

The experiences reported show that this approach is technically sound, clinically relevant, and culturally effective. TIMA not only enhances the quality of synthetic data, but also promotes a new paradigm of collaboration between medicine and technology, which is essential for the safe and informed integration of AI into hospital practice.

Ultimately, TIMA represents a replicable framework that can help define clinical quality standards for AI in cardiology, supporting the transition from theoretical solutions to tools that are genuinely usable in everyday care.

## Data Availability

The original contributions presented in this study are included in the article. Further inquiries can be directed to the corresponding author.
